# A Whispering Heart: Viral Myocarditis Hiding in Plain Sight

**DOI:** 10.7759/cureus.86684

**Published:** 2025-06-24

**Authors:** Khaja Shafiuddin, Noor Sadiq Syed, Trang Singhal

**Affiliations:** 1 Emergency Medicine, The Mid Yorkshire National Health Service (NHS) Teaching Trust, Wakefield, GBR

**Keywords:** cardio vascular disease, emergency medicine resuscitation, fulminant myocarditis, heart blocks, paediatric clinical cardiology

## Abstract

We present the case of a 14-year-old girl with a background of celiac disease and no other significant medical history who developed acute complete heart block and cardiogenic shock secondary to fulminant myocarditis. She presented with persistent vomiting and lethargy. An electrocardiogram (ECG) revealed ST-segment elevation*. *A bedside echocardiogram revealed global hypokinesia. She was found to be in complete heart block and showed biochemical evidence of multi-organ hypoperfusion. Management included emergency inotropic support, hyperkalemia treatment, and extracorporeal membrane oxygenation (ECMO). She made a remarkable recovery, with near normalization of cardiac function and resolution of arrhythmias. This case highlights the importance of high clinical suspicion for myocarditis in pediatric patients presenting with conduction abnormalities and shock.

## Introduction

Myocarditis is a rare but potentially life-threatening inflammatory condition of the myocardium, particularly in the pediatric population. Clinical presentation can range from mild symptoms to fulminant heart failure and arrhythmias. Complete heart block (CHB) is an uncommon manifestation but may signify severe myocardial involvement. Early diagnosis and prompt multidisciplinary management are crucial to improving outcomes [1-3].

## Case presentation

A 14-year-old girl with known celiac disease presented to the emergency department with a one-day history of persistent vomiting (approximately 15-20 episodes), profound fatigue, and clamminess. She reported a brief viral illness two weeks prior to presentation. On examination, she appeared pale, hypotensive (blood pressure 85/42 mmHg), and bradycardic (heart rate 40-60 bpm), with prolonged capillary refill and weak peripheral pulses. She was alert and oriented (Glasgow Coma Scale (GCS) 15).

**Figure 1:**
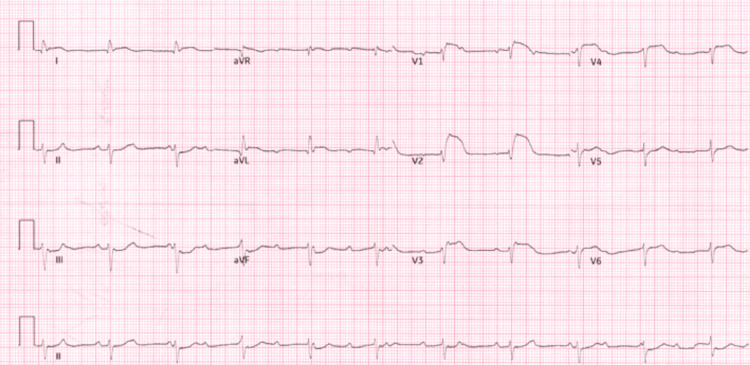
Electrocardiograph demonstrating ST-elevation in leads V1-V4 and ST-depression in the inferior leads

**Figure 2:**
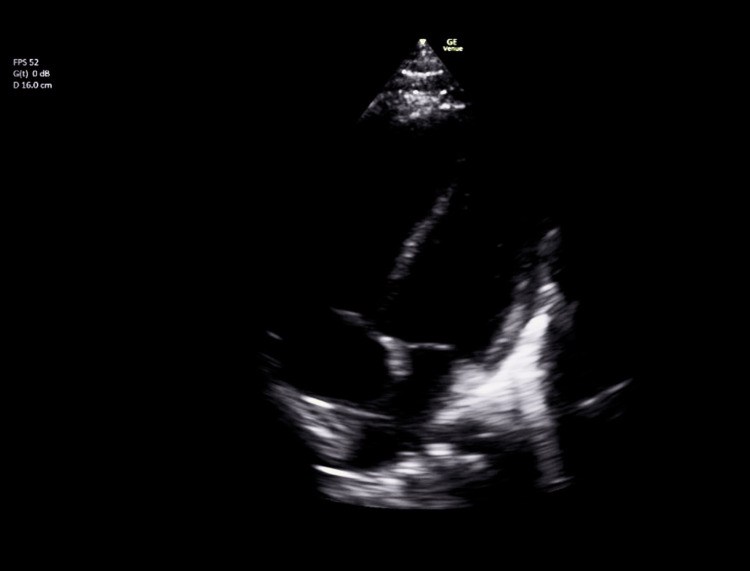
Point-of-care ultrasound demonstrating global hypokinesia

Electrocardiography (ECG) (Figure 1) revealed CHB with ST-elevation in leads V1-V4 and ST-depression in the inferior leads. Point-of-care echocardiography (Figure 2) demonstrated global hypokinesia. Capillary blood (Table 1) gas showed compensated metabolic acidosis (pH 7.45, base excess -9.4, bicarbonate (HCO_3_) 11.8), elevated lactate (8.7 mmol/L), hyperkalemia (K+ 7.5 mmol/L), and hyponatremia (Na+ 129 mmol/L). Cardiac biomarkers were markedly elevated, with a troponin I level of 8506 ng/L.

**Table 1:** Venous blood gas analysis

Parameter	Result	Unit	Normal Range
pH	7.45	-	7.35 - 7.45
pCO₂	2.3	kPa	4.7 - 6.0
pO₂	12.3	kPa	10 - 13
Sodium (Na⁺)	129	mmol/L	135 - 145
Potassium (K⁺)	7.5	mmol/L	3.5 - 5.0
Calcium (Ca²⁺)	1.25	mmol/L	2.1 - 2.6
Glucose (Glu)	incalc	mmol/L	3.9 - 5.8
Lactate (Lac)	8.7	mmol/L	< 2.0
Total Hb (tHb)	—	-	120 - 180 g/L
Oxyhemoglobin (O₂Hb)	96.7	%	95 - 98%
Carboxyhemoglobin (COHb)	1.7	%	< 2%
Methemoglobin (MetHb)	0.6	%	< 1.5%
Deoxyhemoglobin (HHb)	1.0	%	< 2%
Oxygen Saturation (sO₂)	99.0	%	> 94%
Base Excess (BE(B))	-9.4	mmol/L	-2 to +2
Bicarbonate (HCO₃⁻(c))	11.8	mmol/L	22 - 28
Standard Bicarb (HCO₃⁻ std)	17.6	mmol/L	22 - 28
Hematocrit (Hct(c))	41	%	36 - 50%

Initial management included calcium gluconate, insulin-dextrose infusion, magnesium sulfate, and low-dose peripheral adrenaline infusion. An arterial line was placed, and arrangements were made for urgent transfer to a tertiary pediatric cardiology center via the Embrace transport team. Following further assessment, she was diagnosed with fulminant viral myocarditis. Due to severe biventricular dysfunction and ongoing cardiogenic shock, veno-arterial extracorporeal membrane oxygenation (ECMO) was initiated. During ECMO support, the patient regained sinus rhythm, and echocardiographic parameters progressively improved. A residual atrial septal defect (iatrogenic from ECMO) was noted. She recovered well and was discharged with a loop recorder in situ and on a stable regimen of bisoprolol and spironolactone.

## Discussion

This case illustrates the importance of maintaining a high index of suspicion for myocarditis in children presenting with nonspecific systemic symptoms and cardiac conduction abnormalities. Myocarditis may present subtly, but fulminant forms can cause rapid hemodynamic compromise. Electrocardiographic abnormalities such as ST elevation and CHB, especially when coupled with elevated cardiac enzymes and echocardiographic evidence of ventricular dysfunction, should prompt urgent intervention [1,3,4].

In pediatric populations, myocarditis is most often post-viral in etiology. The pathophysiology involves myocardial inflammation and necrosis, leading to impaired contractility and arrhythmogenesis. Timely initiation of mechanical circulatory support such as ECMO can be life-saving and has been associated with favorable neurological and cardiac outcomes [5,6].

The diagnostic workup for suspected myocarditis should include ECG, cardiac biomarkers, echocardiography, and MRI when feasible. Endomyocardial biopsy remains the gold standard but is often reserved for select cases [2,7]. Long-term follow-up is essential to monitor for recurrent arrhythmias or late-onset cardiomyopathy [3,8].

## Conclusions

Acute fulminant myocarditis should be considered in any child presenting with arrhythmia, shock, or unexplained cardiac dysfunction. Prompt ECG interpretation and bedside echocardiography are essential for diagnosis. Early multidisciplinary intervention, including ECMO, can significantly improve patient outcomes and facilitate complete recovery.
